# Exploration of M2 macrophage-related biomarkers and a candidate drug for glioblastoma using high-dimensional weighted gene co-expression network analysis

**DOI:** 10.3389/fphar.2025.1587258

**Published:** 2025-06-30

**Authors:** Suyin Feng, Long Zhu, Jinyuan Gu, Kun Kou, Yongtai Liu, Guangmin Zhang, Hua Lu, Honglai Zhang, Runfeng Sun

**Affiliations:** ^1^ Department of Neurosurgery, Affiliated Hospital of Jiangnan University, Wuxi, China; ^2^ Department of Neurosurgery, Donghai County People’s Hospital, Donghai, China; ^3^ Jiangnan University Smart Healthcare Joint Laboratory, Donghai County People’s Hospital, Donghai, China; ^4^ Cardio-Cerebral Vascular Disease Prevention and Treatment Innovation Center, Donghai County People’s Hospital, Donghai, China; ^5^ Donghai Intelligent Medical Innovation Center, Kangda College of Nanjing Medical University, Lianyungang, China

**Keywords:** tumor-associated macrophages, M2 macrophages, glioblastoma, high-dimensional weighted gene co-expression network analysis, thalidomide

## Abstract

**Background:**

Macrophages exhibit diverse activation states. Notably, M2 macrophages, alternatively activated cells, are notably increased within glioblastoma (GBM). Herein, our current study aimed to identify gene biomarkers relevant to M2 macrophages using high-dimensional weighted gene co-expression network analysis (hdWGCNA) and predict a candidate drug for GBM.

**Methods:**

Single-cell RNA sequencing (scRNA-seq) data (GSE162631) and expression data (GSE4290) for GBM were obtained from the Gene Expression Omnibus (GEO) database. The Seurat package was used for quality control, processing of scRNA-seq data, and identification of different GBM cell types. Subsequently, the clusterProfiler package was employed to functionally annotate the genes specifically highly expressed in the cells. Notably, genes related to the M2 macrophages were screened by differential expression analysis, and the gene modules were classified by hdWGCNA. Thereafter, a diagnostic model was constructed, and its robustness was tested. Moreover, drug candidates that could bind to the specific genes identified in this study were predicted and further confirmed via molecular docking.

**Results:**

Ten cell clusters were classified, with macrophages showing a higher proportion in GBM samples. Moreover, highly expressed genes specific to the M2 macrophages were mainly enriched in neutrophil migration, myeloid leukocyte migration, and chemokine production. A total of 11 gene modules (module 1–11) specific to M2 macrophages were also determined; notably, module 7 showed a relatively high expression of genes. Three key genes, namely, nuclear factor-kappa-B-inhibitor alpha (*NFKBIA*), nuclear receptor 4A2 (*NR4A2*), and FosB Proto-Oncogene, AP-1 Transcription Factor Subunit (*FOSB*), were obtained by intersecting 3,257 differentially expressed genes (DEGs) with the hub genes screened by hdWGCNA. These three genes were applied to establish a robust and reliable diagnostic model, and they were found to bind to the candidate drug thalidomide.

**Conclusion:**

The current study revealed the potential gene biomarkers and drug candidate for GBM based on genes related to M2 macrophages, contributing to the understanding of the underlying mechanism of GBM.

## 1 Introduction

Glioblastoma (GBM) is an aggressive and incurable brain tumor that accounts for approximately 49% of all malignant brain tumors (Zhou et al., 2024; Liu et al., 2024; Guo et al., 2024). The current standard treatments mainly include surgery and radio-chemotherapy using alkylating agents and the supplementation of tumor-treating fields (Tan et al., 2020; Weller et al., 2021). The prognosis of GBM remains dismal, with an overall survival of 14–21 months (Stupp et al., 2017). A study highlighted the complex functional interactions between GBM and the cellular architecture of the brain, underscoring the intricate relationship between the tumor and the central nervous system (Salvalaggio et al., 2024).

A major limitation of the existing therapies lies in their focus solely on GBM cells while neglecting the dynamic interplay of the tumor with its microenvironment (Quail and Joyce, 2017). Within the microenvironment of GBM, immune cells, particularly tumor-associated macrophages (TAMs), have been widely studied (Jain et al., 2023). TAMs, consisting of microglia- or monocyte-derived populations, are self-renewing populations that exhibit significant heterogeneity and dominate the immune landscape in newly diagnosed tumors (Pombo Antunes et al., 2021). Macrophages can functionally polarize into two phenotypes (M1 and M2); in particular, M2 macrophages inhibit inflammation and are found in increased proportion in cerebral tumors such as GBM (Michiba et al., 2022). Moreover, an animal model experiment revealed that M2 TAMs can be activated and in turn enhance tumor progression under the guidance from tumor-released immunosuppressive cytokines and chemokines, while the breakdown of M2 TAMs effectively suppresses GBM (Louis et al., 2016; Pyonteck et al., 2013; Moghaddam et al., 2023). This evidence, therefore, suggests that M2 TAMs in the tumor microenvironment may be a potential therapeutic target for GBM.

Computational analyses have become indispensable in the screening of tumor-specific genes and prognosis-relevant biomarkers, contributing to the development of cancer therapeutics (Huang et al., 2018; Yan et al., 2025). At present, data from the Gene Expression Omnibus (GEO) database are commonly used to assess gene transcription levels, support the monitoring of mRNA expressions, and predict cellular functions (Yin et al., 2022; Wang et al., 2025). As an unbiased systematic biology analysis method, weighted gene co-expression network analysis (WGCNA) explores co-expressed gene modules (Li et al., 2023a; Wang et al., 2024), whereas high-dimensional weighted gene co-expression network analysis (hdWGCNA) is a comprehensive framework that analyzes the co-expression networks based on high-dimensional transcriptomics data (Morabito et al., 2023). So far, the application of hdWGCNA in GBM remains limited, presenting an opportunity for further exploration. Herein, our current study aimed to identify M2 macrophage-related gene biomarkers for GBM and screen effective drug candidates that bind to the biomarkers via molecular docking. The goal of the present study was to reveal the mechanisms underlying the involvement of M2 macrophages in GBM and provide some novel insights for this field.

## 2 Methods

### 2.1 Data source

The single-cell RNA sequencing (scRNA-seq) data were extracted from the dataset GSE162631, which contained four GBM samples. The chip data of GBM were obtained from the dataset GSE4290, which contained 77 GBM samples and 23 normal samples from epilepsy patients.

### 2.2 Processing the scRNA-seq data

The scRNA-seq data were first read using the “Seurat” R package to retain the cells with the following criteria: 1) mitochondrial gene count between 200 and 6,000 and 2) mitochondrial gene count >10% (Tan et al., 2023). Thereafter, the data were standardized using the SCTransform function and subjected to dimensionality reduction via principal component analysis (PCA). The “harmony” R package was applied to remove the batch effects to ensure a robust downstream analysis. For dimensionality reduction analysis, we performed uniform manifold approximation and projection (UMAP) using the first 50 principal components (PCs). Subsequently, a k-nearest neighbor (KNN) plot was generated based on the Euclidean distance using the FindNeighbors function. Finally, all the cells were clustered via the FindCluster function at the resolution of 0.1 (for the macrophages, the resolution was set at 0.05) and annotated using the known marker genes provided by the CellMarker database (Xu et al., 2024).

### 2.3 Identification and functional enrichment analysis of higher-expression genes

Specifically higher-expression genes in different cell clusters were identified using the “FindAllMarkers” function at the following parameters: logfc.threshold = 0.30, min.pct = 0.25, and only.pos = T. The functional enrichment analysis on these genes was implemented using the “clusterProfiler” R package (Yu et al., 2012).

### 2.4 Identification of M2 macrophage-related biomarkers

The hdWGCNA is a systems biology analysis method for identifying co-expressed gene modules and mining key regulatory factors based on high-dimensional transcriptomic data. In this study, the rds data were read using the “hdWGCNA” R package (Morabito et al., 2023). The co-expression network was established on M2 macrophages under the selected optimal soft threshold to obtain relevant gene modules. The correlation between the gene modules and M2 macrophages was calculated to reveal the gene module of interest. The connectivity of the gene modules was determined to identify the hub genes in the module.

### 2.5 Identification of the DEGs

All the samples were divided into the control and GBM groups, and differential gene expression was calculated for the two groups using the “Limma” R package (Ritchie et al., 2015). The relevant DEGs were subsequently filtered under the criteria of |log_2_FC| ≥ 1 and the adjusted *p*-value <0.01.

Then, the obtained DEGs were intersected with the hub genes identified by hdWGCNA to obtain the key gene biomarkers for GBM.

### 2.6 Construction of a diagnostic model

To quantify the risk for GBM patients, a nomogram was established with the key gene biomarkers using the “rms” R package (Li et al., 2023b). The predictive potential and the robustness of the nomogram were then tested based on the receiver operator characteristics (ROC) curve (and the calculated area under the curve (AUC) value), calibration curve, and decision curve.

### 2.7 Candidate drug prediction for GBM and molecular docking

Enrichment analysis on the gene set was implemented via the “Enrichr” R package (Kuleshov et al., 2016). Based on the dataset DSigDB, the binding of the candidate drug to the key gene biomarkers was predicted. The crystal structure of the receptor proteins was then obtained from the UniProt database, prioritizing those determined by X-ray or NMR, with lower-resolution structures serving as the secondary source. The positions were extended long enough to ensure a comprehensive coverage of the adequate binding sites.

Based on the prediction results, the 3D structure of the drug candidate was downloaded from PubChem as the ligand, and the drug candidates were scaled down according to their *p*-value if they did not have any 3D structure or required gene overexpression or knockdown. The PyMOL software was utilized to remove water molecules and small molecules and add hydrogen molecules. The energy of the 3D structure of the drug candidate was minimized by using the ChemBioOffice software, and molecular docking was performed to obtain the results with binding energy <−5 kcal/mol and hydrogen bond length <3.5 Å. The detailed parameters for each molecular docking are listed in Table 1 (Xiao et al., 2024).

**TABLE 1:** Parameters for molecular docking in this assay.

Term	Spacing	Npts	Center
FOSB_thalidomide	0.747	40 40 126	3.334 −0.885 −21.310
NFKBIA_thalidomide	0.681	126 126 126	38.520 25.943 28.220
NR4A2_thalidomide	0.642	126 126 126	17.362 −40.441 −16.738

### 2.8 Statistical analysis

All computational analyses of this study were realized in R software 4.1.1. The data of two groups were compared using the Wilcoxon test. The threshold of statistical significance was set when the *p*-value was below 0.05.

## 3 Results

### 3.1 Single-cell landscape in GBM

**FIGURE 1:**
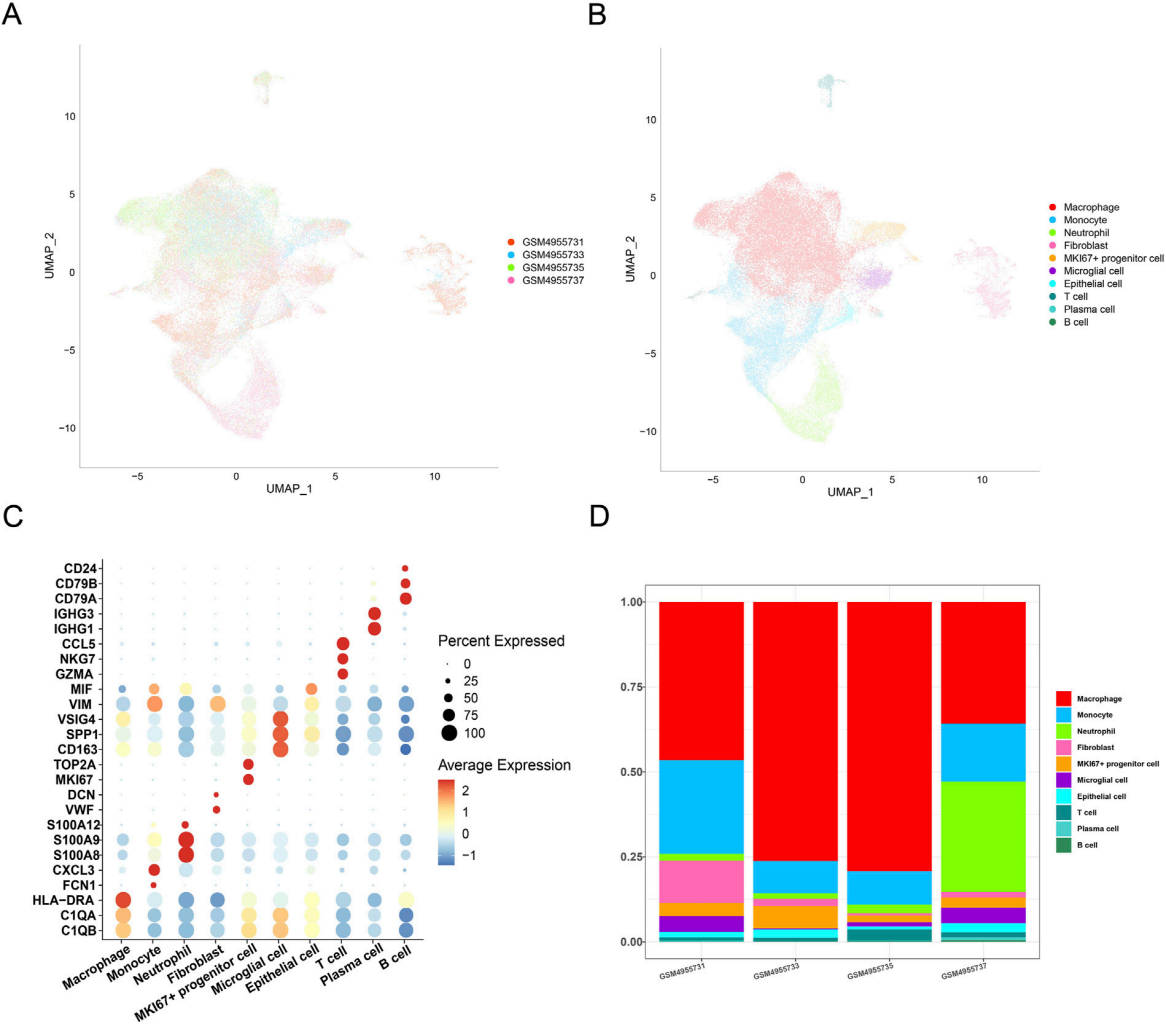
Single-cell landscape in GBM. **(A)** UMAP plot showing the distribution of samples following the removal of batch effects. **(B)** UMAP plot displaying different cell types in GBM. **(C)** Expression levels of marker genes belonging to different cell types in GBM. **(D)** Percentage of different cell types in different GBM samples.

The scRNA-seq analysis was performed to classify cell clusters of GBM. Following data filtering, standardization, removal of batch effects, and dimensionality reduction, the cells were divided into 10 main clusters (Figures 1A,B). Based on the annotation from the CellMarker2.0 database, the following cell types were defined: macrophages (C1QB, C1QA, and HLA-DRA), monocytes (FCN1 and CXCL3), neutrophils (S100A8, S100A9, and S100A12), fibroblasts (VWF and DCN), MKI67^+^ progenitor cells (MKI67 and TOP2A), microglial cells (CD163, SPP1, and VSIG4), epithelial cells (VIM and MIF), T cells (GZMA, NKG7, and CCL5), plasma cells (IGHG1, IGHG3, and CD79A), and B cells (CD79A, CD79B, and CD24). The expression levels of the marker genes in these cell clusters are displayed in Figure 1C. Calculation of the percentage of the 10 clusters in the four GBM samples revealed a relatively higher percentage of macrophages (Figure 1D). These discoveries indicated the potential involvement of macrophages in GBM.

### 3.2 Landscape of macrophages in GBM

**FIGURE 2:**
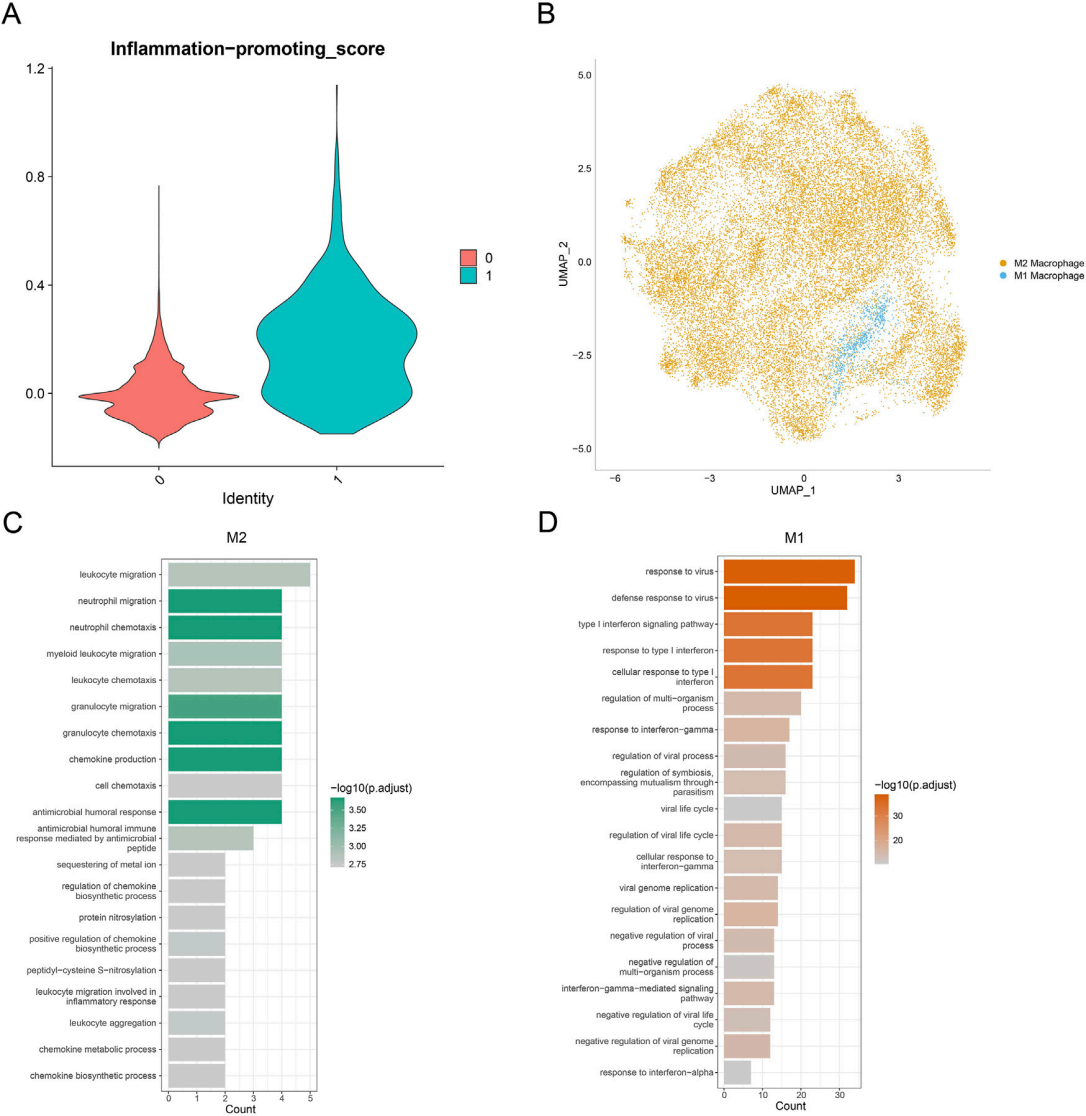
Landscape of the macrophage subpopulation in GBM. **(A)** Violin plot on the inflammation-promoting score of macrophage subpopulations. **(B)** Distribution on the macrophage subpopulations based on the visualization using the UMAP plot. **(C,D)** Bar chart displaying the top 20 enriched items of genes specifically highly expressed in macrophage subpopulations.

To characterize macrophage subpopulations in GBM, they were divided into two main subclusters. Considering the dynamic transformation of M1 and M2 macrophages, it is difficult to distinguish these subclusters using the markers. Hence, AddModuleScore was applied to calculate the score of the pro-inflammatory factors (Figure 2A). An evidently higher score was seen in cluster_1 than in cluster_0. Accordingly, cluster_1 was marked as M1 macrophages, and cluster_0 was marked as M2 macrophages (Figure 2B).

Subsequently, highly expressed genes specific to M1 and M2 macrophages were subjected to Gene Ontology (GO) enrichment analysis. It was observed that the genes associated with M2 macrophages were mainly enriched in neutrophil migration, myeloid leukocyte migration, and chemokine production, whereas the genes related to M1 macrophages were mainly enriched in the defense response to virus regulation or viral life cycle and negative regulation of viral genome replication (Figures 2C,D). Collectively, these results provided the preliminary data for the polarization tendency of macrophages in GBM and the potential enriched pathways of their specific genes.

### 3.3 Identifying M2 macrophage-related gene modules via hdWGCNA

**FIGURE 3:**
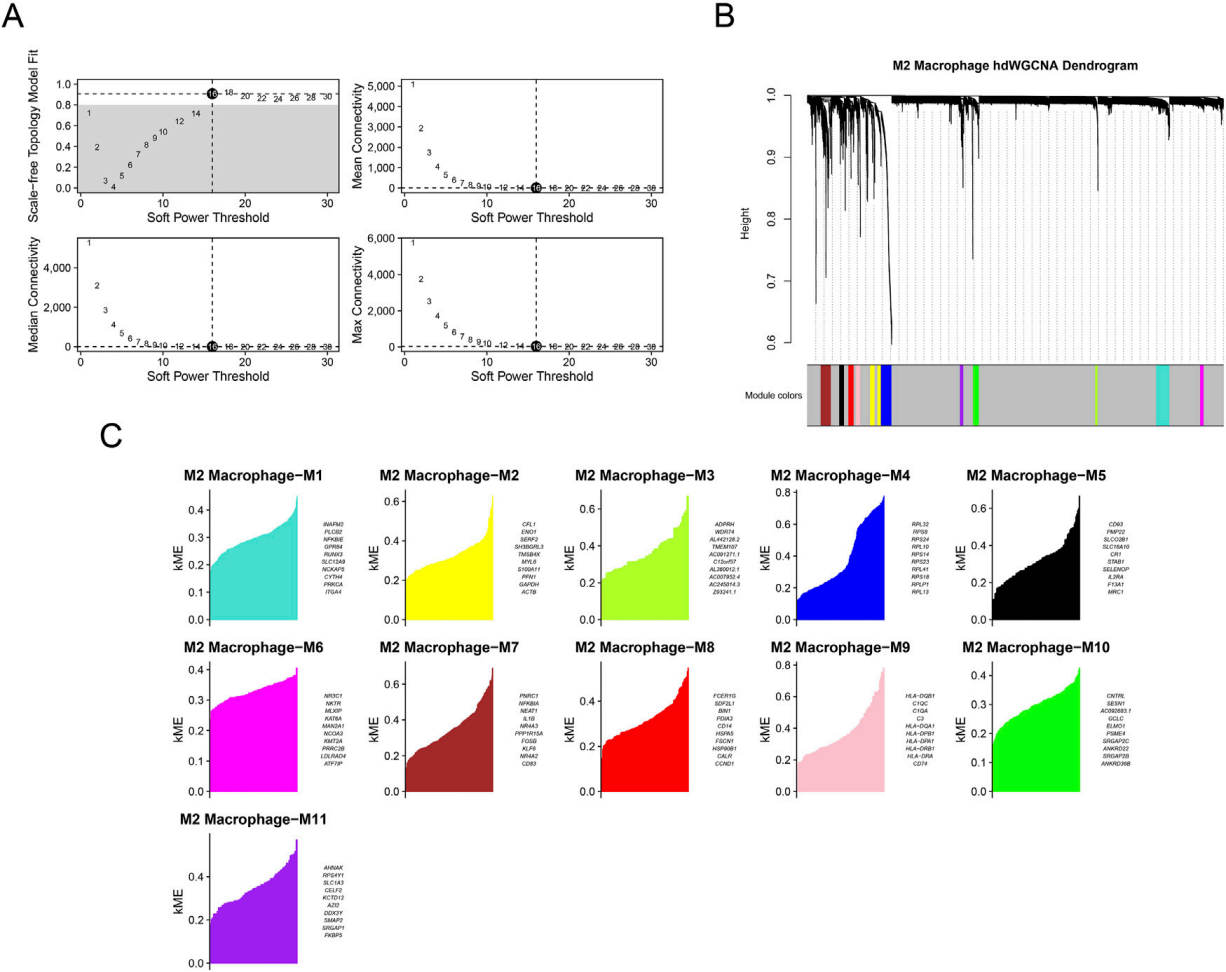
Sorting of M2 macrophage-related gene modules via hdWGCNA. **(A)** Sorting of the optimal soft power threshold for hdWGCNA. **(B)** Dendrogram of hdWGCNA of M2 macrophages. **(C)** Division of gene modules (M2 macrophage-M1 to M2 macrophage-M11) for hdWGCNA.

Then, M2 macrophage-related gene modules were classified using hdWGCNA. Based on the computed optimal soft power threshold of 16 (Figure 3A), we constructed a gene clustering dendrogram in the hdWGCNA framework with M2 macrophages as the study object. Different colored bars at the bottom indicated a total of 11 modules identified, each consisting of genes with similar expression patterns (Figure 3B). The eigengene-based connectivity kME was further calculated to reveal 11 gene modules (M2 macrophage-M1 to M2 macrophage-M11 (Figure 3C)).

**FIGURE 4:**
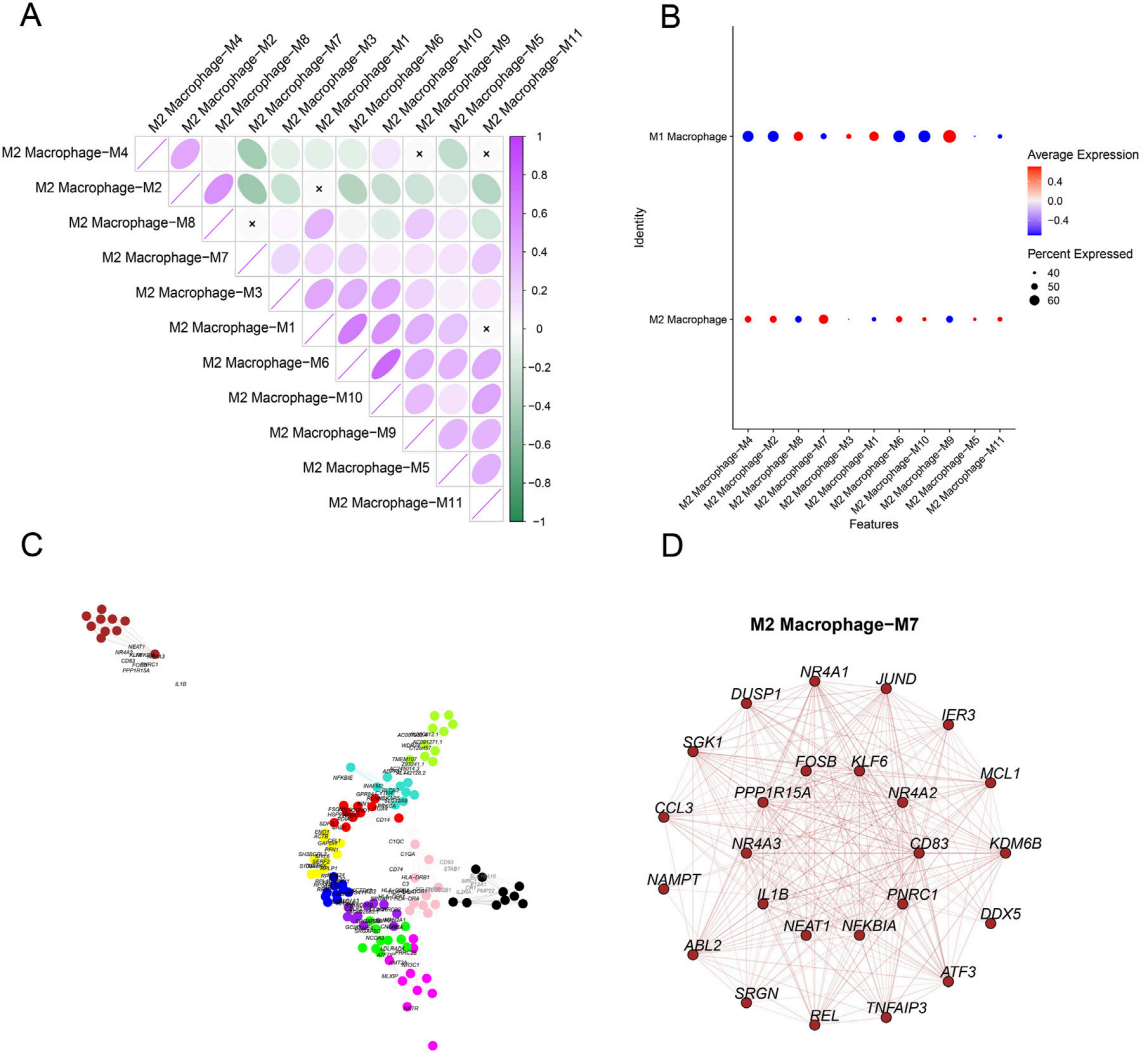
Identification of M2 macrophage-specific gene modules based on hdWGCNA. **(A)** Expression level of genes belonging to each module in different cells (red represents the highly expressed genes, and blue represents the lower expressed genes). **(B)** Correlation matrix in different gene modules. **(C)** Co-expression network of hub genes belonging to each gene module. **(D)** Co-expression network of the hub gene based on gene module M2 macrophage-M7.

The expression levels of genes in the modules in different cell clusters were quantified, showing a relatively high expression of genes in M2 macrophage-M7 (Figure 4A). Therefore, M2 macrophage-M7 was regarded as the key module for plotting the correlation matrix (Figure 4B) and gene co-expression network (Figure 4C). The hub gene network of the M2 macrophage-M7 module was additionally generated based on the 10 primary key genes (in the inner circle) and the 15 secondary key genes (in the outer circle), as shown in Figure 4D.

### 3.4 Analysis on the DEGs based on GEO data

**FIGURE 5:**
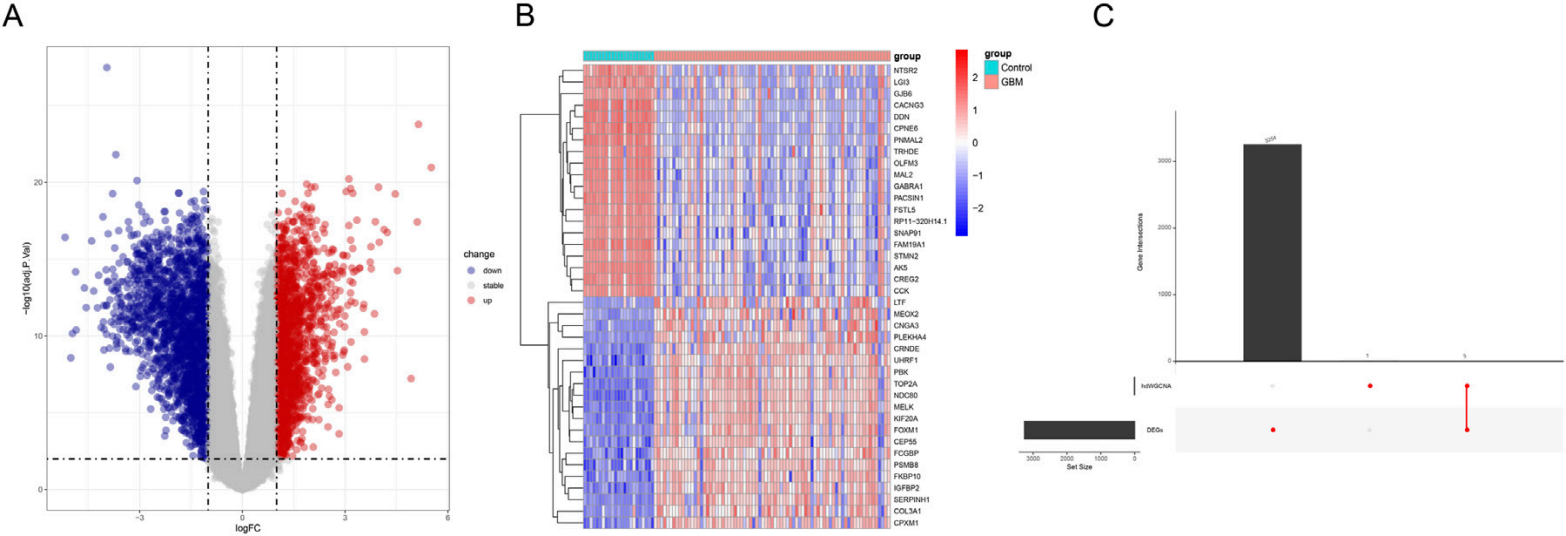
Analysis of the differentially expressed genes based on GEO data. **(A)** Volcano plot showing the differentially expressed genes based on GEO data (red represents the up-regulated differentially expressed genes, and blue represents the down-regulated differentially expressed genes). **(B)** Heatmap demonstrating the expression levels of the differentially expressed genes (red represents the up-regulated differentially expressed genes, and blue represents the down-regulated differentially expressed genes). **(C)** Upset plot on the differentially expressed genes and the hub gene from hdWGCNA.

The samples of GBM and control from GEO data were utilized to screen the DEGs under the thresholds of |log_2_fold change| ≥ 1 and adjusted *p*-value < 0.01. In total, 3,257 DEGs (1,459 up-regulated DEGs and 1,798 down-regulated genes) were identified (Figure 5A). The top 20 up-regulated and down-regulated DEGs were selected to draw a heatmap. As shown in Figure 5B, some genes (e.g., *IGFBP2*, *CPOL3A1*, and *CEP55*) were highly expressed in GBM. Subsequently, we acquired three common genes (*NR4A2, NR4A2,* and *FOSB)* by intersecting the DEGs with the hub genes identified by hdWGCNA (Figure 5C).

### 3.5 Construction of a diagnostic model

**FIGURE 6:**
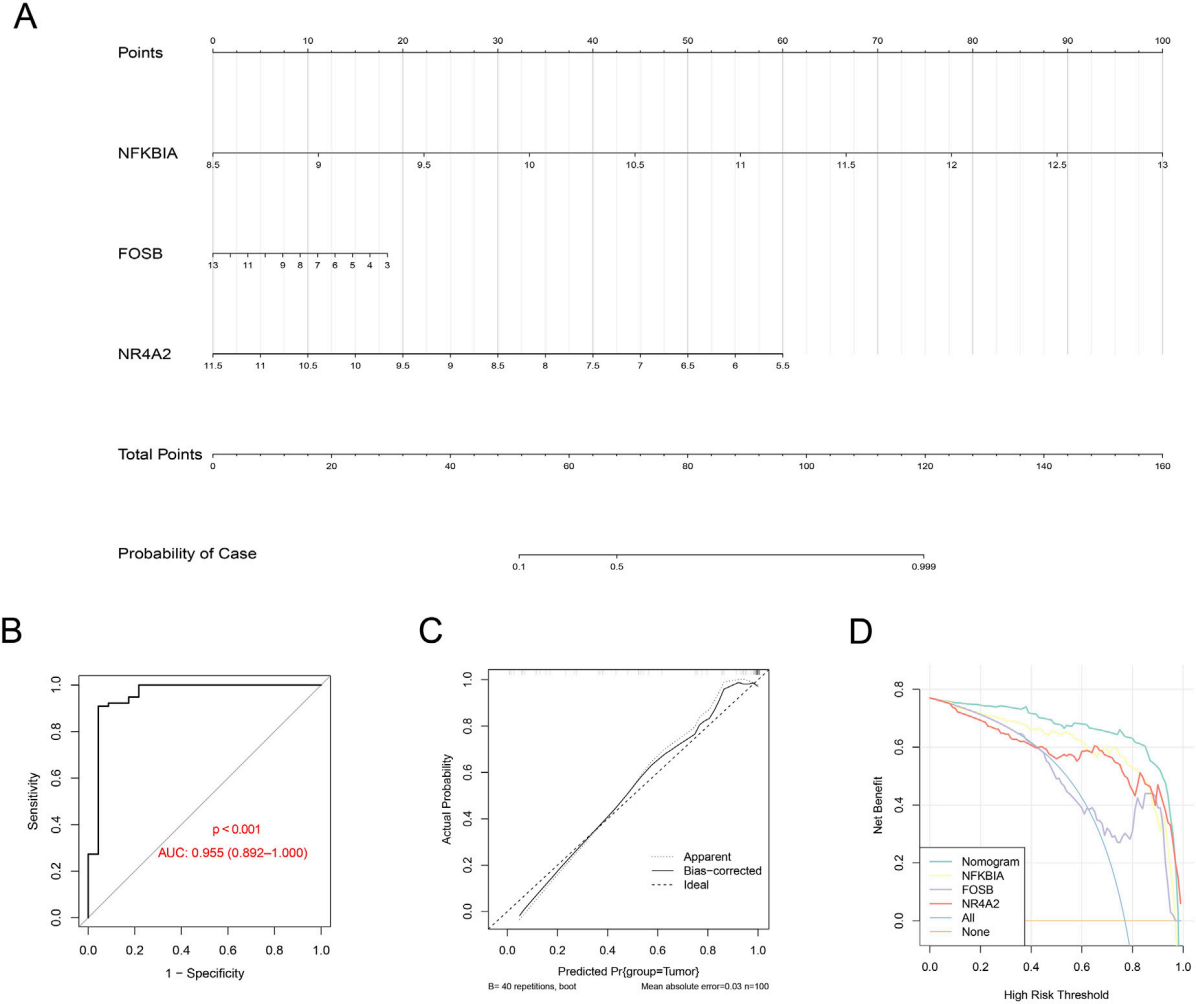
Construction of the diagnostic model based on the hub genes. **(A)** Nomogram using the gene expression level of the hub genes (with the corresponding score based on the expression level). **(B–D)** ROC curve **(B)**, calibration curve **(C)**, and decision curve **(D)** based on the established nomogram.

A nomogram was created using the expression levels of these three key genes to quantify the risk for GBM patients (Figure 6A). The nomogram showed an AUC = 0.955 (Figure 6B), and the calibration curve and the ROC curve were nearly overlaid (Figure 6C), suggesting a high predictive efficacy of the diagnostic model. Furthermore, the decision curve was plotted to evaluate the robustness of the model, and an evidently higher benefit of the nomogram than that of a single gene was noticed (Figure 6D). These discoveries collectively demonstrated a strong predictive value of our diagnostic model.

### 3.6 Prediction of the drug candidate for GBM and molecular docking

**FIGURE 7:**
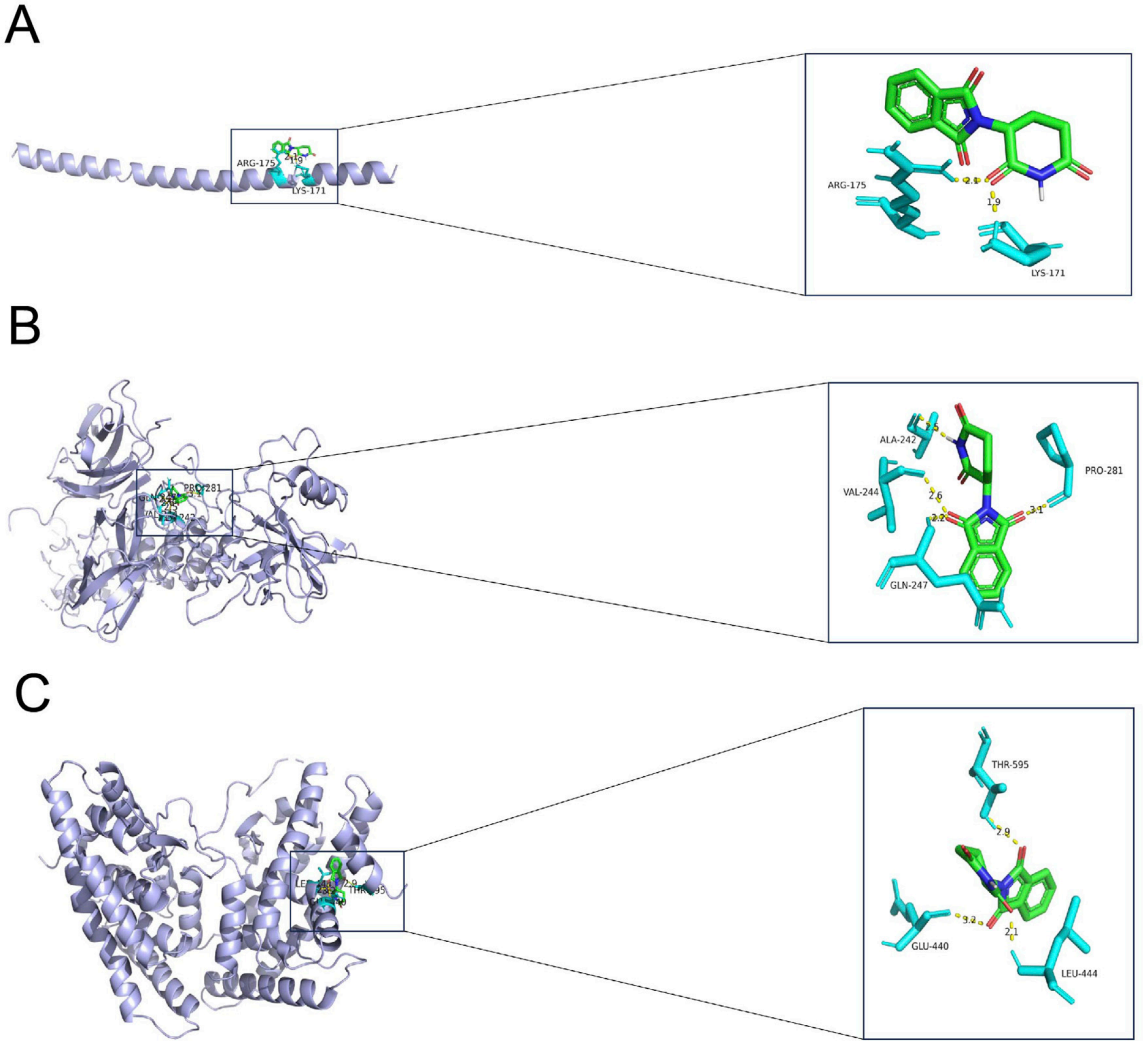
Result of molecular docking showing the drug candidates. **(A–C)** Molecular docking results showing the docking of thalidomide with FOSB **(A)**, NFKBIA **(B)**, and NR4A2 **(C)**. Blue molecules in the figure represent the receptor proteins, the green molecules represent the drug molecules, and the turquoise molecules represent the amino acid. The binding between the receptor proteins and the drug molecules is shown in the form of a yellow dotted line. The number in the figure is the length of the hydrogen bond (Å).

Finally, the three key genes were uploaded and analyzed via the Enrichr package, and the binding of the drug candidates to the three genes was predicted using the DSigDB database. Finally, thalidomide was predicted as the potential drug for GBM, and 6ucl, 1ikn, and 5y41 were the protein structures for *FOSB*, *NFKB1A,* and *NR4A2*, respectively. Notably, the protein structures of the three genes all stably bound to thalidomide (Figures 7A–C; Table 2).

**TABLE 2:** The binding energy based on the molecular docking.

Compound CID	Molecular_name	Gene_name	PDB_ID	Energy (kcal/mol)
5,426	thalidomide	FOSB	6ucl	−5.14
5,426	thalidomide	NFKBIA	1ikn	−7.46
5,426	thalidomide	NR4A2	5y41	−7.31

## 4 Discussion

Macrophages have been considered scavengers that regulate the immune response against pathogens and maintain homeostasis within tissue. In response to various cytokine stimulations, macrophages undergo a switch in their metabolic pathways, which lead to their differentiation into either the inflammatory (M1) or regulatory (M2) subtypes (Mehla and Singh, 2019). While TAMs do not strictly follow M1/M2 polarization, they generally exhibit an M2-like polarization state to facilitate the growth of tumors via triggering immune suppression (Mehla and Singh, 2019). Tumor–immune cell interactions are increasingly recognized as critical drivers of GBM progression and invasion. In particular, the crosstalk between TAMs and GBM cells promotes an immunosuppressive microenvironment that facilitates tumor growth, angiogenesis, and resistance to therapy (Hambardzumyan et al., 2015). M2-polarized TAMs can secrete a variety of cytokines and growth factors, such as TGF-β and VEGF, which contribute to extracellular matrix remodeling and enhance tumor invasiveness (Pombo Antunes et al., 2021; Li et al., 2022; Peng et al., 2022). These findings encouraged us to further explore biomarkers related to M2 TAMs in the research of GBM. Understanding these complex interactions not only deepens our knowledge of GBM pathobiology but also paves the way for developing personalized therapeutic strategies targeting specific immune components. Following the identification of M2 macrophages, specifically highly expressed genes were found to be enriched in the pathways such as neutrophil migration, myeloid leukocyte migration, and chemokine production. Existing studies have demonstrated that neutrophil migration plays a critical role in initiating and enhancing the inflammatory response by facilitating the recruitment of immune cells to the sites of tissue injury or infection (de Oliveir et al., 2016). Moreover, neutrophils, which play a crucial part in the innate immune system, can also promote the growth of GBM cells (Wang et al., 2023; Chen et al., 2022). Circulating myeloid cells refer to mature neutrophils and monocytes, which can migrate out from the blood vessels and into the tissue in response to inflammation (Marelli-Berg and Jangani, 2018). In addition, some prior studies have shown the association between some chemokines such as CC chemokine ligand 1 (CCL1) and M2 macrophage polarization (Sironi et al., 2006). These findings indicated a potential link between the highly expressed genes in M2 macrophages and the pathways related to inflammatory cell migration and chemokine activity, which requires further investigation in the context of GBM.

This study identified the gene modules specific to M2 macrophages in GBM using hdWGCNA. Earlier studies on GBM have applied WGCNA to discover anoikis- and prognosis-related genes (Sun et al., 2022; Zhou et al., 2021; Sun et al., 2024). Similarly, some other investigations employed WGCNA to classify M2 macrophage-related gene modules in chronic rhinosinusitis with nasal polyps (Zhu et al., 2022), proliferative diabetic retinopathy (Meng et al., 2022), and melanoma (Wu et al., 2022). Based on the analysis of hdWGCNA, we revealed 11 gene modules linked to M2 macrophages in GBM; in particular, the genes in module M7 were notably highly expressed in M2 macrophages. The hub genes of the module M7 were accordingly selected to be intersected with the DEGs screened from both the control and tumor groups. Finally, *NFKBIA*, *NR4A2*, and *FOSB* were determined as three common M2 macrophage-related genes in GBM. *NFKBIA* has been identified as an inhibitor of nuclear factor-kappa B (NF-κB) and exerts an anti-tumor effect on GBM (Komotar et al., 2011). *NR4A2* has been extensively characterized in the cerebral subcellular regions and is indispensable for the normal function of dopaminergic neurons. A study also supported that *NR4A2* could be a druggable target of GBM (Karki et al., 2020). *FOSB* is reported as an oncogene in GBM, and knockdown of *FOSB* could inhibit the growth of GBM cells *in vitro* and *in vivo* (Qi et al., 2022). In the current study, the three common genes, which bound to the drug candidate thalidomide (a cancer treatment drug with anti-inflammatory, immuno-modulatory, and anti-angiogenic properties and some neuroprotective effect on adults), were applied to establish a robust and reliable diagnostic model (Franks et al., 2004; Vargesson and Stephens, 2021). Some other studies have also demonstrated the potential therapeutic effects of thalidomide in treating GBM (Eatmann et al., 2023; Hassler et al., 2015) and that thalidomide intervention leads to altered perfusion and permeability of GBM (Conq et al., 2024). Therefore, we speculated that the three common genes may be the druggable targets of thalidomide in GBM, and our future study will continue to validate the speculation.

Some limitations in the present research should be pointed out. Since the sample size for the analysis was relatively small, some larger cohorts should be incorporated to better validate the generalization of the present study results. Second, the study was an *in silico* analysis without any laboratory or clinical validation. Randomized clinical trials or relevant experimental analyses should be added for further verification. Third, while we particularly focused on M2 macrophage-related key genes in researching the biomarkers for GBM, some other cell clusters may be potentially equally important. Thus, our future study will continue to explore the specific implications of these cell clusters and relevant specific genes to provide a better insight into the molecular mechanisms of GBM.

## 5 Conclusion

To conclude, our study identified M2 macrophage-related gene biomarkers via hdWGCNA and predicted a potential candidate drug for GBM, providing valuable insights into the potential molecular mechanisms underlying the progression of GBM. These computational analyses can be applied as the prediction tools to evaluate the prognostic outcomes for patients and facilitate the selection of appropriate immunotherapy strategies.

### 5.1 Scope statement

Ten cell clusters were identified, and a higher percentage of macrophages was seen in GBM samples. Moreover, the highly expressed genes specific to M2 macrophages were mainly enriched in neutrophil migration, myeloid leukocyte migration, and chemokine production. A total of 11 gene modules specific to M2 macrophages were also classified, and a relatively high expression of genes in module 7 in M2 macrophages was noted. The DEGs were intersected with the hub genes from hdWGCNA to obtain three key genes for GBM, namely, *NFKBIA*, *NR4A2*, and *FOSB*. These three genes were used to establish a robust and reliable diagnostic model, and they bound to the drug candidate thalidomide. The current study revealed the potential gene biomarkers and drug candidates for GBM based on M2 macrophages, contributing to the understanding of the underlying mechanisms of GBM.

## Data Availability

The datasets presented in this study can be found in online repositories. The names of the repository/repositories and accession number(s) can be found in the article/supplementary material.

## Abbreviations

GBM, glioblastoma; TAMs, tumor-associated macrophages; GEO, gene expression omnibus; WGCNA, weighted gene co-expression network analysis; hdWGCNA, high-dimensional WGCNA; scRNA-seq, single-cell RNA sequencing; PCA, principal component analysis; UMAP, uniform manifold approximation and projection; DEGs, differentially expressed genes; ROC, receiver operator characteristics; AUC, area under the curve; GO, Gene Ontology; NFKBIA, nuclear factor-kappa-B-inhibitor alpha; NR4A2, nuclear Receptor 4A2; FOSB, FosB proto-oncogene, AP-1 transcription factor subunit; CCL1, CC chemokine ligand 1; NF-κB, nuclear factor-kappa B; PCs, principal components.
